# Crystal structure of (*E*)-2-fluoro­benz­aldehyde (pyridin-2-yl)hydrazone

**DOI:** 10.1107/S2056989015007823

**Published:** 2015-04-30

**Authors:** Haliwana B. V. Sowmya, Tholappanavara H. Suresha Kumara, Jerry P. Jasinski, Sean P. Millikan, Christopher Glidewell

**Affiliations:** a PG Department of Chemistry, Jain University, 52 Bellary Road, Hebbal, Bangalore 560 024, India; b University B.D.T. College of Engineering (a Constituent College of VTU, Belgaum), Davanagere 577 004, India; c Department of Chemistry, Keene State College, 229 Main Street, Keene, NH 03435-2001, USA; d School of Chemistry, University of St Andrews, St Andrews, Fife KY16 9ST, Scotland

**Keywords:** crystal structure, hydrazine, hydrogen bonding, π–π stacking inter­actions

## Abstract

The title compound, C_12_H_10_FN_3_, is approximately planar: the dihedral angles between the mean plane of the central N—N=C spacer unit and the fluoro­benzene and pyridine rings are 14.50 (13) and 4.85 (15)°, respectively, while the dihedral angle between the aromatic rings is 16.29 (6)°. The F atom lies at the same side of the mol­ecule as the N atom of the pyridine ring. In the crystal, inversion dimers linked by pairs of N—H⋯N hydrogen bonds generate *R*_2_^2^(8) loops. Mol­ecules related by translation in the *a* direction are linked by two π–π stacking inter­actions involving pairs of benzene rings and pairs of pyridine rings. In each case, the ring-centroid separation is 3.8517 (9) Å. Two chains of this type pass through each unit cell, but there are no direction-specific inter­actions between adjacent chains.

## Related literature   

For crystal structures of related hydrazones, see: Ferguson *et al.* (2005 ▸); Wardell *et al.* (2005 ▸); Gomes *et al.* (2013 ▸).
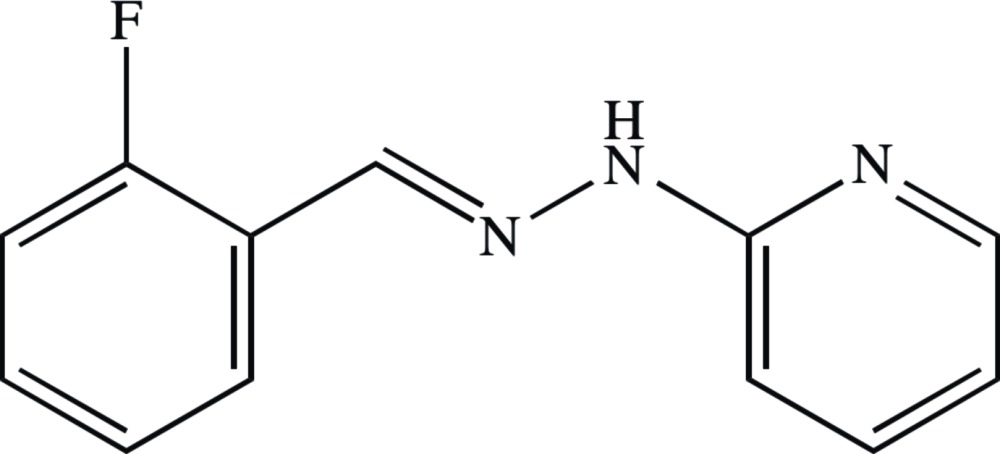


## Experimental   

### Crystal data  

C_12_H_10_FN_3_
*M* *_r_* = 215.23
Monoclinic, 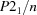

*a* = 3.85166 (14) Å
*b* = 23.1757 (7) Å
*c* = 11.4227 (4) Å
β = 99.278 (4)°
*V* = 1006.31 (6) Å^3^
*Z* = 4
Cu *K*α radiation
μ = 0.84 mm^−1^
*T* = 173 K
0.42 × 0.35 × 0.16 mm

### Data collection  

Agilent Eos Gemini CCD diffractometer
Absorption correction: multi-scan (*CrysAlis RED*; Agilent, 2012 ▸) *T*_min_ = 0.419, *T*_max_ = 0.875
6087 measured reflections
1962 independent reflections
1774 reflections with *I* > 2σ(*I*)
*R*_int_ = 0.033

### Refinement  

*R*[*F* ^2^ > 2σ(*F* ^2^)] = 0.044
*wR*(*F* ^2^) = 0.122
*S* = 1.09
1962 reflections
148 parameters
H atoms treated by a mixture of independent and constrained refinement
Δρ_max_ = 0.21 e Å^−3^
Δρ_min_ = −0.26 e Å^−3^

### 

Data collection: *CrysAlis PRO* (Agilent, 2012 ▸); cell refinement: *CrysAlis PRO*; data reduction: *CrysAlis RED* (Agilent, 2012 ▸); program(s) used to solve structure: *SHELXS97* (Sheldrick, 2008 ▸); program(s) used to refine structure: *SHELXL2014* (Sheldrick, 2015 ▸); molecular graphics: *PLATON* (Spek, 2009 ▸); software used to prepare material for publication: *SHELXL2014* and *PLATON*.

## Supplementary Material

Crystal structure: contains datablock(s) global, I. DOI: 10.1107/S2056989015007823/hb7410sup1.cif

Structure factors: contains datablock(s) I. DOI: 10.1107/S2056989015007823/hb7410Isup2.hkl

Supporting information file. DOI: 10.1107/S2056989015007823/hb7410Isup3.cml

**Fig. 1.**
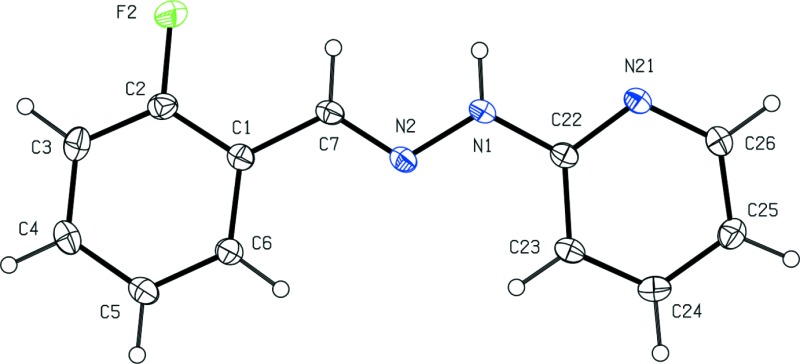
The molecular structure of the title compound showing displacement ellipsoids drawn at the 30% probability level.

**Fig. 2.**
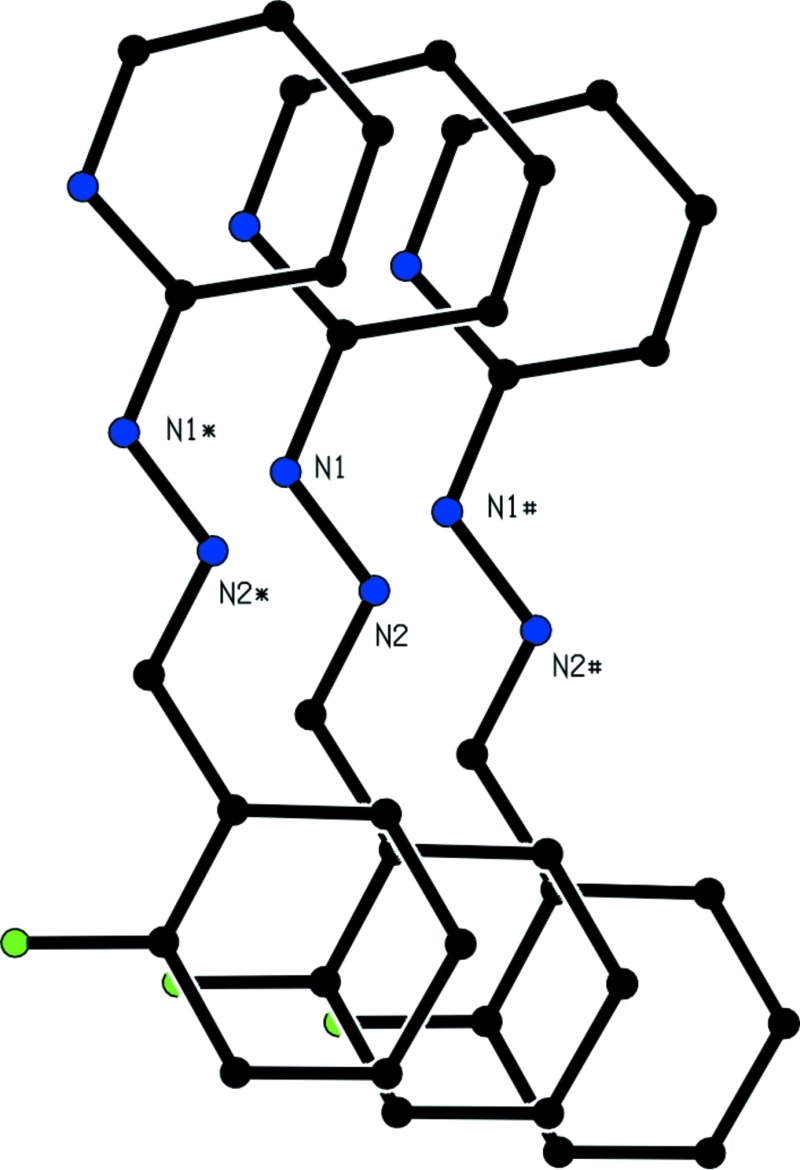
Part of the crystal structure of the title compound showing the π-overlap between molecules related by translation along [100]. For the sake of clarity, the unit-cell outline and the H atoms have been omitted. The atoms marked with an asterisk (*) or a hash (#) are at the symmetry positions (-1 + x, y, z) and) 1 + x, y, z) respectively.

. DOI: 10.1107/S2056989015007823/hb7410fig1.tif
The mol­ecular structure of the title compound showing displacement ellipsoids drawn at the 30% probability level.

x y z x y z . DOI: 10.1107/S2056989015007823/hb7410fig2.tif
Part of the crystal structure of the title compound showing the π-overlap between mol­ecules related by translation along [100]. For the sake of clarity, the unit-cell outline and the H atoms have been omitted. The atoms marked with an asterisk (*) or a hash (#) are at the symmetry positions (−1 + *x*, *y*, *z*) and) 1 + *x*, *y*, *z*) respectively.

**Fig. 3.**
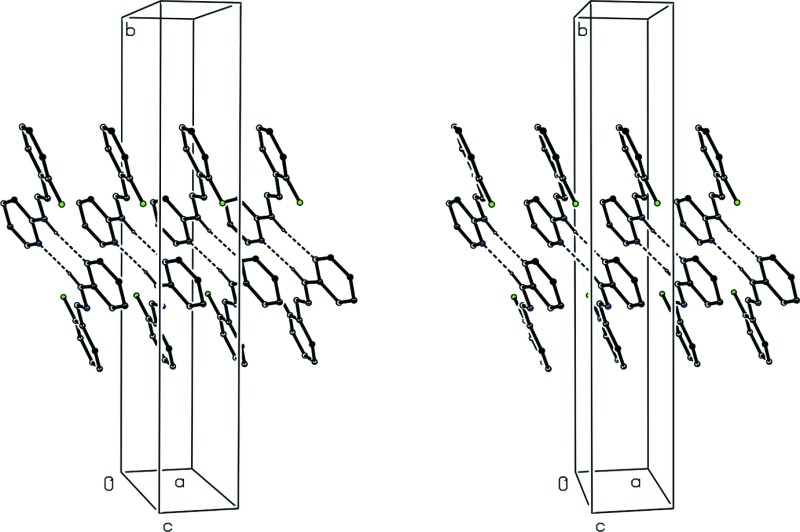
A stereoview of part of the crystal structure of the title compound showing the formation of a π-stacked chain of hydrogen-bonded dimers. For the sake of clarity the H atoms bonded to C atoms have been omitted.

. DOI: 10.1107/S2056989015007823/hb7410fig3.tif
A stereoview of part of the crystal structure of the title compound showing the formation of a π-stacked chain of hydrogen-bonded dimers. For the sake of clarity the H atoms bonded to C atoms have been omitted.

CCDC reference: 1060682

Additional supporting information:  crystallographic information; 3D view; checkCIF report

## Figures and Tables

**Table 1:** Hydrogen-bond geometry (, )

*D*H*A*	*D*H	H*A*	*D* *A*	*D*H*A*
N1H1N21^i^	0.90(2)	2.21(2)	3.1020(17)	174.1(19)

Symmetry code: (i) 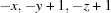.

## supplementary crystallographic information

### S1. Structural commentary

For pairs of aryl rings in molecules related by translation along [100], the ring centroid separation is 3.8517 (9) Å and the inter­planar spacing is 3.5178 (6) Å corresponding to a ring-centroid offset of 1.568 (2) Å; for an analogous pair of pyridyl rings, the corresponding values are 3.8516 (8) Å, 3.3347 (6) Å, and 1.927 (2) Å respectively. Despite the presence of two independent rings and a large excess of C—H bonds, there are no C—H···π hydrogen bonds in the crystal structure.

### S2. Synthesis and crystallization

2-Pyridyl­hydrazine (439.5 mg, 4.0 mmol) was added to a solution of 2-fluoro­benzaldehyde (500 mg, 4.0 mmol) in methanol (10 ml) and stirred for *ca.* 2 min. The progress of the reaction was monitored by TLC. After completion, water (10 ml) was added and the resulting solid was collected by filtration, washed with water, dried, and crystallized by slow evaporation, at ambient temperature of a solution in methanol to give the product in the form of colourless plates in essentially qu­anti­tative yield.

### S3. Refinement

Crystal data, data collection and structure refinement details are summarized in Table 1. All H atoms were located in difference maps. The H atoms bonded to C atoms were then treated as riding atoms in geometrically idealized positions with C—H distances 0.95 Å and *U*_iso_(H) = 1.2*U*_eq_(C). For the H atom bonded to atom N1, the atomic coordinates were refined with *U*_iso_(H) = 1.2*U*_eq_(N) giving an N—H distance of 0.90 (2) Å.

### Crystal data

**Table:**

C_12_H_10_FN_3_	*F*(000) = 448
*M**_r_* = 215.23	*D*_x_ = 1.421 Mg m^−^^3^
Monoclinic, *P*2_1_/*n*	Cu *K*α radiation, λ = 1.54184 Å
*a* = 3.85166 (14) Å	Cell parameters from 1962 reflections
*b* = 23.1757 (7) Å	θ = 3.8–72.5°
*c* = 11.4227 (4) Å	µ = 0.84 mm^−^^1^
β = 99.278 (4)°	*T* = 173 K
*V* = 1006.31 (6) Å^3^	Plate, colourless
*Z* = 4	0.42 × 0.35 × 0.16 mm

### Data collection

**Table:**

Agilent Eos Gemini CCD diffractometer	1774 reflections with *I* > 2σ(*I*)
Radiation source: Enhance (Cu) X-ray Source	*R*_int_ = 0.033
ω scans	θ_max_ = 72.5°, θ_min_ = 3.8°
Absorption correction: multi-scan (*CrysAlis RED*; Agilent, 2012)	*h* = −4→4
*T*_min_ = 0.419, *T*_max_ = 0.875	*k* = −20→28
6087 measured reflections	*l* = −13→13
1962 independent reflections	

### Refinement

**Table:**

Refinement on *F*^2^	0 restraints
Least-squares matrix: full	Hydrogen site location: structure-invariant direct methods
*R*[*F*^2^ > 2σ(*F*^2^)] = 0.044	H atoms treated by a mixture of independent and constrained refinement
*wR*(*F*^2^) = 0.122	*w* = 1/[σ^2^(*F*_o_^2^) + (0.0674*P*)^2^ + 0.2786*P*] where *P* = (*F*_o_^2^ + 2*F*_c_^2^)/3
*S* = 1.09	(Δ/σ)_max_ < 0.001
1962 reflections	Δρ_max_ = 0.21 e Å^−^^3^
148 parameters	Δρ_min_ = −0.26 e Å^−^^3^

### Special details

**Table:**

Geometry. All e.s.d.'s (except the e.s.d. in the dihedral angle between two l.s. planes) are estimated using the full covariance matrix. The cell e.s.d.'s are taken into account individually in the estimation of e.s.d.'s in distances, angles and torsion angles; correlations between e.s.d.'s in cell parameters are only used when they are defined by crystal symmetry. An approximate (isotropic) treatment of cell e.s.d.'s is used for estimating e.s.d.'s involving l.s. planes.

### Fractional atomic coordinates and isotropic or equivalent isotropic displacement parameters (Å^2^)

**Table:**

	*x*	*y*	*z*	*U*_iso_*/*U*_eq_	
C1	0.3610 (4)	0.33820 (6)	0.24399 (13)	0.0248 (3)	
C2	0.3174 (4)	0.34404 (6)	0.12182 (13)	0.0298 (3)	
F2	0.1734 (3)	0.39368 (4)	0.07270 (8)	0.0469 (3)	
C3	0.4131 (4)	0.30233 (7)	0.04731 (13)	0.0348 (4)	
H3	0.3812	0.3085	−0.0360	0.042*	
C4	0.5569 (4)	0.25120 (7)	0.09603 (14)	0.0332 (4)	
H4	0.6231	0.2217	0.0463	0.040*	
C5	0.6032 (4)	0.24350 (6)	0.21772 (14)	0.0318 (4)	
H5	0.7017	0.2085	0.2513	0.038*	
C6	0.5076 (4)	0.28620 (6)	0.29089 (13)	0.0286 (3)	
H6	0.5419	0.2802	0.3742	0.034*	
C7	0.2600 (4)	0.38495 (6)	0.31780 (13)	0.0265 (3)	
H7	0.1313	0.4170	0.2814	0.032*	
N21	0.2532 (3)	0.48299 (5)	0.65928 (11)	0.0266 (3)	
C22	0.3541 (3)	0.43452 (6)	0.61006 (12)	0.0242 (3)	
C23	0.5634 (4)	0.39220 (6)	0.67501 (13)	0.0274 (3)	
H23	0.6310	0.3583	0.6375	0.033*	
C24	0.6673 (4)	0.40114 (6)	0.79396 (14)	0.0314 (3)	
H24	0.8084	0.3732	0.8403	0.038*	
C25	0.5667 (4)	0.45118 (7)	0.84726 (13)	0.0330 (4)	
H25	0.6370	0.4582	0.9296	0.040*	
C26	0.3618 (4)	0.49000 (6)	0.77589 (13)	0.0299 (3)	
H26	0.2923	0.5242	0.8117	0.036*	
N1	0.2387 (3)	0.42916 (5)	0.49067 (11)	0.0291 (3)	
H1	0.107 (5)	0.4566 (9)	0.4499 (16)	0.035*	
N2	0.3428 (3)	0.38325 (5)	0.43070 (11)	0.0265 (3)	

### Atomic displacement parameters (Å^2^)

**Table:**

	*U*^11^	*U*^22^	*U*^33^	*U*^12^	*U*^13^	*U*^23^
C1	0.0216 (6)	0.0228 (7)	0.0294 (7)	−0.0021 (5)	0.0019 (5)	−0.0018 (5)
C2	0.0307 (7)	0.0254 (7)	0.0314 (8)	−0.0022 (6)	−0.0006 (6)	0.0025 (6)
F2	0.0711 (7)	0.0331 (5)	0.0331 (5)	0.0088 (4)	−0.0016 (5)	0.0062 (4)
C3	0.0390 (8)	0.0388 (9)	0.0263 (7)	−0.0070 (6)	0.0039 (6)	−0.0049 (6)
C4	0.0303 (8)	0.0314 (8)	0.0387 (8)	−0.0027 (6)	0.0078 (6)	−0.0110 (6)
C5	0.0299 (7)	0.0250 (7)	0.0397 (8)	0.0033 (5)	0.0033 (6)	−0.0026 (6)
C6	0.0293 (7)	0.0259 (7)	0.0299 (7)	0.0020 (5)	0.0031 (6)	−0.0007 (5)
C7	0.0269 (7)	0.0199 (7)	0.0319 (7)	0.0021 (5)	0.0030 (6)	0.0008 (5)
N21	0.0288 (6)	0.0205 (6)	0.0306 (6)	0.0005 (4)	0.0054 (5)	−0.0002 (4)
C22	0.0232 (7)	0.0200 (7)	0.0300 (7)	−0.0022 (5)	0.0061 (5)	0.0015 (5)
C23	0.0256 (7)	0.0208 (7)	0.0361 (8)	0.0005 (5)	0.0056 (6)	0.0017 (5)
C24	0.0269 (7)	0.0301 (8)	0.0361 (8)	0.0023 (6)	0.0014 (6)	0.0068 (6)
C25	0.0318 (8)	0.0388 (8)	0.0275 (7)	−0.0006 (6)	0.0020 (6)	−0.0010 (6)
C26	0.0312 (7)	0.0271 (7)	0.0319 (8)	−0.0004 (6)	0.0062 (6)	−0.0044 (6)
N1	0.0361 (7)	0.0208 (6)	0.0294 (6)	0.0078 (5)	0.0028 (5)	−0.0010 (5)
N2	0.0277 (6)	0.0203 (6)	0.0314 (6)	0.0017 (4)	0.0042 (5)	−0.0019 (4)

### Geometric parameters (Å, º)

**Table:**

C1—C2	1.385 (2)	N21—C26	1.3401 (19)
C1—C6	1.400 (2)	N21—C22	1.3416 (18)
C1—C7	1.4634 (19)	C22—N1	1.3702 (18)
C2—F2	1.3586 (17)	C22—C23	1.4032 (19)
C2—C3	1.377 (2)	C23—C24	1.369 (2)
C3—C4	1.386 (2)	C23—H23	0.9500
C3—H3	0.9500	C24—C25	1.394 (2)
C4—C5	1.384 (2)	C24—H24	0.9500
C4—H4	0.9500	C25—C26	1.375 (2)
C5—C6	1.383 (2)	C25—H25	0.9500
C5—H5	0.9500	C26—H26	0.9500
C6—H6	0.9500	N1—N2	1.3604 (16)
C7—N2	1.2782 (19)	N1—H1	0.90 (2)
C7—H7	0.9500		
			
C2—C1—C6	116.45 (13)	C26—N21—C22	116.93 (12)
C2—C1—C7	120.53 (13)	N21—C22—N1	115.04 (12)
C6—C1—C7	123.02 (13)	N21—C22—C23	122.98 (13)
F2—C2—C3	118.12 (13)	N1—C22—C23	121.97 (12)
F2—C2—C1	118.28 (13)	C24—C23—C22	118.04 (13)
C3—C2—C1	123.60 (14)	C24—C23—H23	121.0
C2—C3—C4	118.77 (14)	C22—C23—H23	121.0
C2—C3—H3	120.6	C23—C24—C25	120.14 (13)
C4—C3—H3	120.6	C23—C24—H24	119.9
C5—C4—C3	119.47 (14)	C25—C24—H24	119.9
C5—C4—H4	120.3	C26—C25—C24	117.33 (14)
C3—C4—H4	120.3	C26—C25—H25	121.3
C6—C5—C4	120.72 (14)	C24—C25—H25	121.3
C6—C5—H5	119.6	N21—C26—C25	124.57 (13)
C4—C5—H5	119.6	N21—C26—H26	117.7
C5—C6—C1	120.99 (14)	C25—C26—H26	117.7
C5—C6—H6	119.5	N2—N1—C22	119.82 (12)
C1—C6—H6	119.5	N2—N1—H1	118.8 (12)
N2—C7—C1	120.82 (12)	C22—N1—H1	121.3 (12)
N2—C7—H7	119.6	C7—N2—N1	115.99 (12)
C1—C7—H7	119.6		
			
C6—C1—C2—F2	179.47 (13)	C26—N21—C22—N1	−179.75 (12)
C7—C1—C2—F2	−1.1 (2)	C26—N21—C22—C23	0.1 (2)
C6—C1—C2—C3	−0.7 (2)	N21—C22—C23—C24	0.1 (2)
C7—C1—C2—C3	178.66 (14)	N1—C22—C23—C24	179.91 (13)
F2—C2—C3—C4	−179.30 (14)	C22—C23—C24—C25	−0.3 (2)
C1—C2—C3—C4	0.9 (2)	C23—C24—C25—C26	0.2 (2)
C2—C3—C4—C5	−0.5 (2)	C22—N21—C26—C25	−0.1 (2)
C3—C4—C5—C6	0.0 (2)	C24—C25—C26—N21	0.0 (2)
C4—C5—C6—C1	0.1 (2)	N21—C22—N1—N2	176.23 (12)
C2—C1—C6—C5	0.2 (2)	C23—C22—N1—N2	−3.6 (2)
C7—C1—C6—C5	−179.17 (13)	C1—C7—N2—N1	179.42 (12)
C2—C1—C7—N2	−170.37 (13)	C22—N1—N2—C7	−171.68 (12)
C6—C1—C7—N2	9.0 (2)		

### Hydrogen-bond geometry (Å, º)

**Table:**

*D*—H···*A*	*D*—H	H···*A*	*D*···*A*	*D*—H···*A*
N1—H1···N21^i^	0.90 (2)	2.21 (2)	3.1020 (17)	174.1 (19)

Symmetry code: (i) −*x*, −*y*+1, −*z*+1.
