# Supplementary figures and images for: Crystal structure of (E)-2-fluoro­benz­aldehyde (pyridin-2-yl)hydrazone
Source: Acta Crystallogr E Crystallogr Commun. 2015 Apr 30;71(Pt 5):o362–3. doi: 10.1107/S2056989015007823 (PMC4420083; doi:10.1107/S2056989015007823)

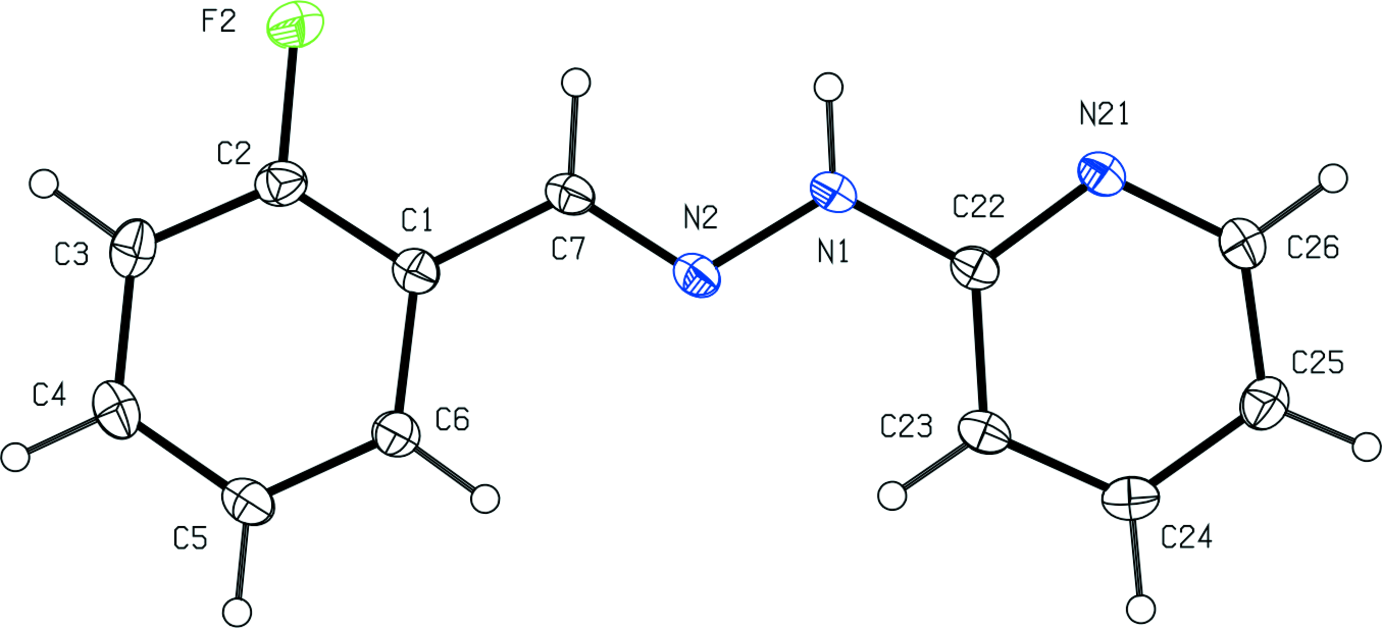

Supplement: Supplementary file 4 [file e-71-0o362-fig1.tif]

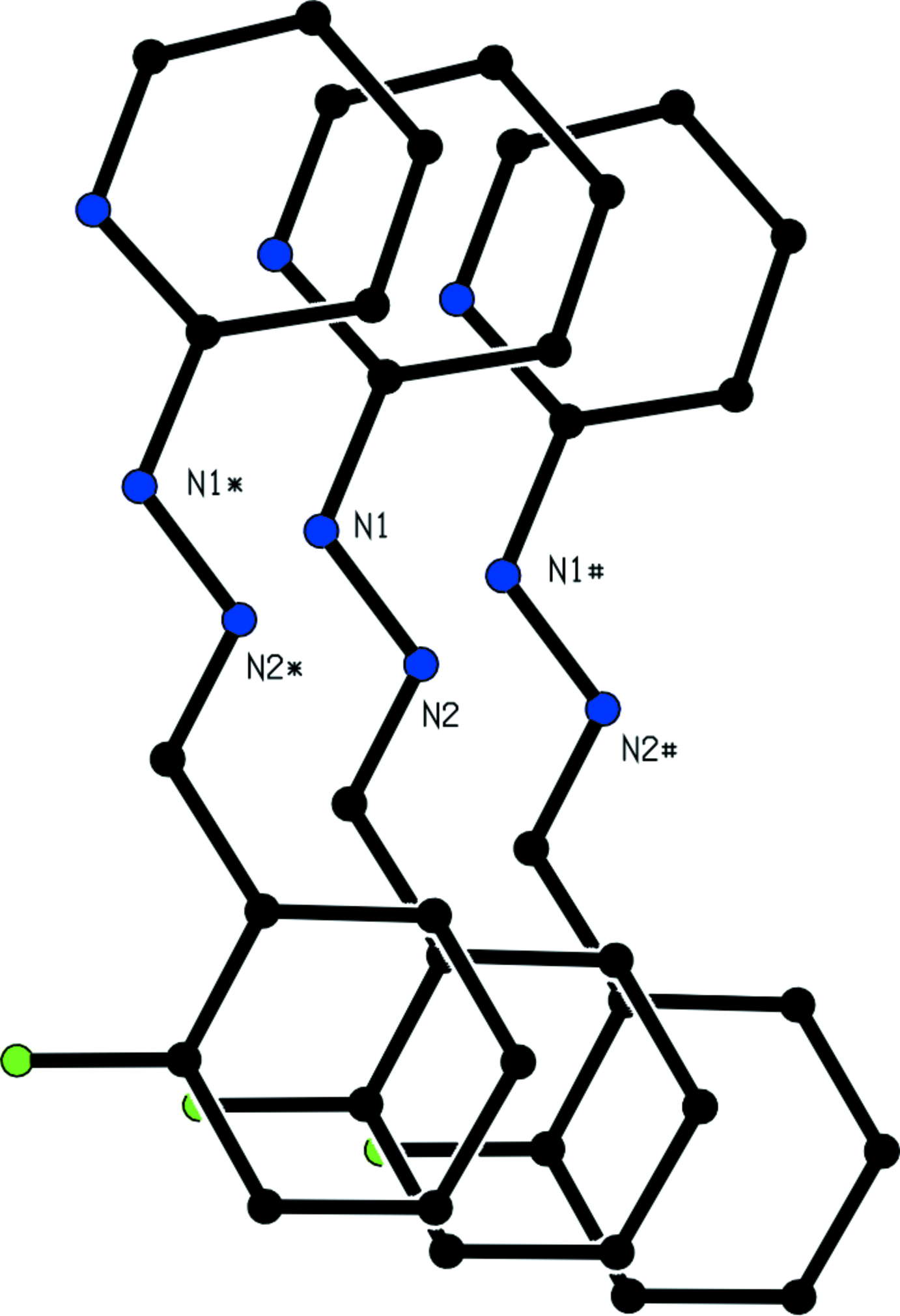

Supplement: Supplementary file 5 [file e-71-0o362-fig2.tif]

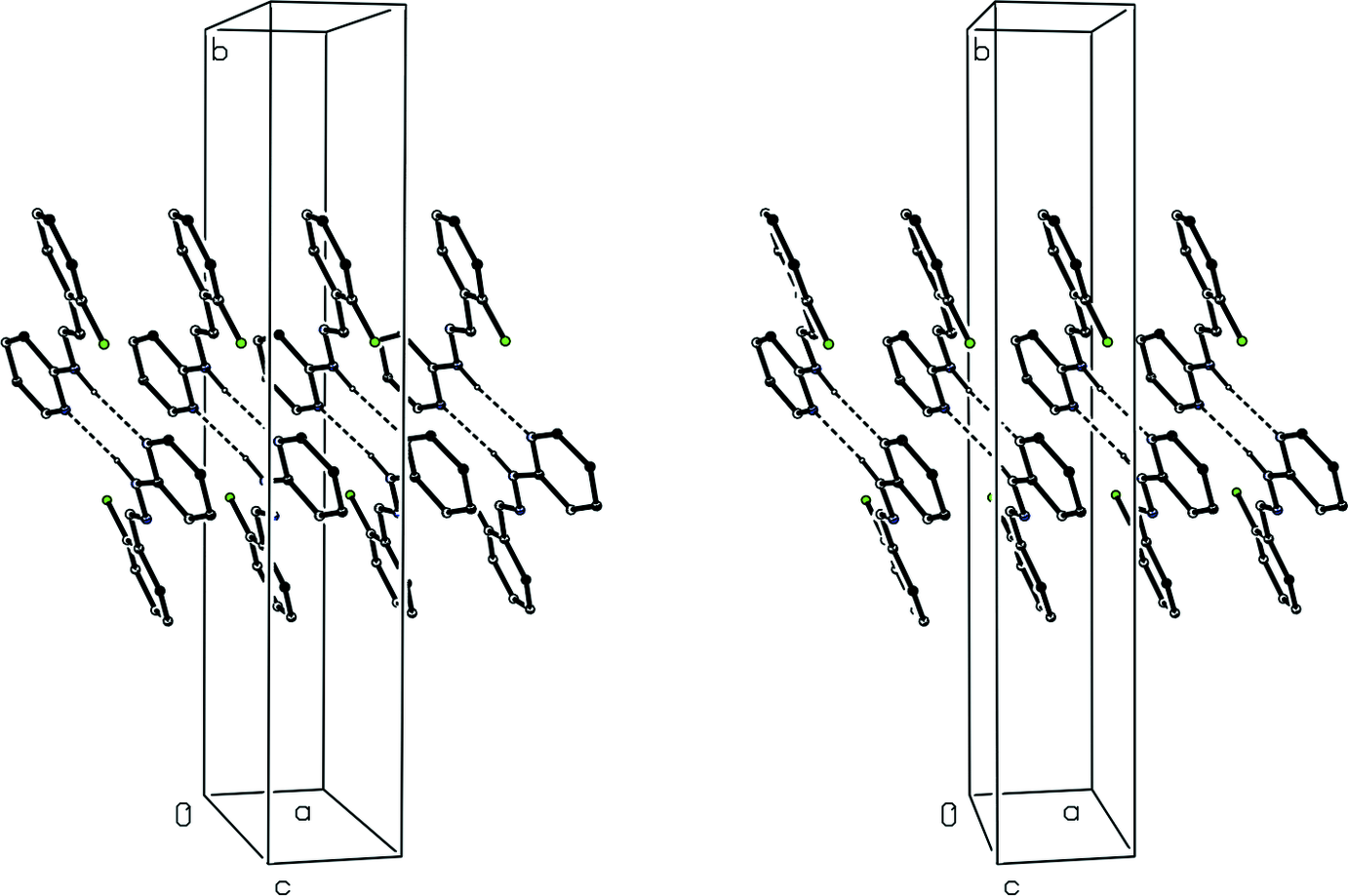

Supplement: Supplementary file 6 [file e-71-0o362-fig3.tif]
